# Structural Comparison of Human Mammalian Ste20-Like Kinases

**DOI:** 10.1371/journal.pone.0011905

**Published:** 2010-08-06

**Authors:** Christopher J. Record, Apirat Chaikuad, Peter Rellos, Sanjan Das, Ashley C. W. Pike, Oleg Fedorov, Brian D. Marsden, Stefan Knapp, Wen Hwa Lee

**Affiliations:** 1 Structural Genomics Consortium, University of Oxford, Oxford, United Kingdom; 2 Nuffield Department of Clinical Medicine, University of Oxford, Oxford, United Kingdom; 3 Department of Clinical Pharmacology, University of Oxford, Oxford, United Kingdom; University of Oulu, Germany

## Abstract

**Background:**

The serine/threonine mammalian Ste-20 like kinases (MSTs) are key regulators of apoptosis, cellular proliferation as well as polarization. Deregulation of MSTs has been associated with disease progression in prostate and colorectal cancer. The four human MSTs are regulated differently by C-terminal regions flanking the catalytic domains.

**Principal Findings:**

We have determined the crystal structure of kinase domain of MST4 in complex with an ATP-mimetic inhibitor. This is the first structure of an inactive conformation of a member of the MST kinase family. Comparison with active structures of MST3 and MST1 revealed a dimeric association of MST4 suggesting an activation loop exchanged mechanism of MST4 auto-activation. Together with a homology model of MST2 we provide a comparative analysis of the kinase domains for all four members of the human MST family.

**Significance:**

The comparative analysis identified new structural features in the MST ATP binding pocket and has also defined the mechanism for autophosphorylation. Both structural features may be further explored for inhibitors design.

**Enhanced version:**

**This article can also be viewed as an enhanced version in which the text of the article is integrated with interactive 3D representations and animated transitions. Please note that a web plugin is required to access this enhanced functionality. Instructions for the installation and use of the web plugin are available in Text S1.**

## Introduction

The Sterile-20 protein kinase (Ste20) was first identified in yeast as a key molecule involved in pheromone signaling [1] and has since been found to be involved in the regulation of a large number of diverse cellular functions including cell polarization [2], [3], actin organization [4], [5], regulation of exit from mitosis [6] and apoptosis [7], [8].

Subsequently several mammalian Ste20-like (MST) kinases sharing homology with the yeast ste20 were identified and grouped into two structurally distinct families: p21-activated kinase (PAK) and the germinal centre kinase (GCK) [9]. The GCK group can be further subdivided into GCKI to GCKVIII [10], [11], [12], [13] and are characterized by the positioning of the kinase domain at the N-terminus, as opposed to the PAK group where the kinase domain can be found at the C-terminus. GCKs are also devoid of an N-terminal GTP-binding domain, found in the PAKs.

The subfamily GCKII includes MST1 and MST2, two upstream kinases implicated in cell growth and apoptosis. Both kinases can be activated via caspase-mediated cleavage of the C-terminal inhibitory domain [14]. Activation of MST1 induces activation of JNK and p38 during apoptosis events in certain cell types [15], [16], [17]. Additionally, MST1 can also induce apoptosis via phosphorylation of histone 2B [7], [18], promotion of chromatin condensation [19], [20] and FOXO3 nuclear translocation in neuronal cells [21]. It has been further found that MST1 acts as an essential mediator in apoptosis of K-ras transformed cells [22], [23] and that the Drosophila homologue Hippo phosphorylates the tumor suppressor protein Salvador and is involved in the regulation of expression of cyclin E and apoptosis inhibitor DIAP1 [24]. Similarly, the human orthologue of Salvador (hSav) can bind and be phosphorylated by both MST1 and 2 [25]. Additionally, MST2 is involved in the LATS tumor suppressor pathway via complexation with hSav, RASSF1A, Nore1 and LATS1, resulting in the phosphorylation of LATS1 and transcription of proapoptotic genes [26], [27], [28].

The subfamily GCKIII contains MST3 and MST4 which share almost 90% amino acid identity in the kinase domain but less than 20% in the C-terminal domain [9]. Despite being related to subfamily II (MST1 and MST2), members of these two groups have apparently different cellular functions. Neither MST3 nor MST4 are capable of activating JNK and p38 MAPK kinase activity [29], [30] and both were shown to activate ERK in different cell lines, albeit via different pathways [29], [31], [32]. In contrast to MST1 and MST2 the C-terminus of MST3 and MST4 stimulates kinase activity by promoting auto-phosphorylation. MST4 has been shown to phosphorylate the actin remodeler Ezrin at its regulatory residue T567 resulting in transmission of cell polarization signals [33].

All MSTs (1–4) are expressed almost ubiquitously in all tissues, with higher expression levels found in placenta (MST1, MST2 and MST4), skeletal tissues (MST1, MST2, MST3), kidney (MST1 and MST2), heart (MST3), pancreas (MST3), thymus, and peripheral blood leukocytes (MST4) [29], [30], [34], [35], [36], [37]. Decreasing levels of MST2 has been observed to correlate with loss of RASSF2 in human colorectal carcinomas [38]. Additionally, MST1 and MST4 have been implicated in prostate cancer, whereby MST1 protein levels declined with disease progression [39] and MST4 expression has been correlated with tumourigenicity of human prostate cancer cell lines [36]. Overexpression of MST4 induces anchorage-independent growth and increases *in vitro* proliferation as well as *in vivo* tumourigenesis in prostate cancer cell lines [40] suggesting a role in prostate cancer progression. MST4 may also be involved in regulation of cell migration via its interaction with the Golgi protein GM130. Binding to GM130 activates MST4 as well as YSK, a related kinase, by promoting autophosphorylation of a conserved threonine within the activation segment. Interference with the kinase function perturbs perinuclear Golgi organization, cell migration, and invasion into type I collagen [41].

Other physiological functions of the MSTs include their possible involvement in the immune system. MST1 is implicated in cardiac apoptosis events (e.g. cardiac dilation, apoptosis and fibrosis after myocardial infarction [42]). MST1 has also been suggested to regulate lymphocyte polarity and adhesion, somatic hypermutation, class switching in B cells and apoptosis of eosinophils (see [9] for review). MST3 has been described to use metal ions other than Mg^2+^ as cofactors for kinase activity, such as Mn^2+^, Zn^2+^ and Co^2+^. Interestingly a variant of MST3 is expressed uniquely in brain, where Mn^2+^ and Zn^2+^ are abundant [32], [43].

Here we present the crystal structure of the unphosphorylated kinase domain of human MST4 refined at 2.35 Å resolution in complex with an ATP mimetic quinazoline inhibitor. We compare the crystal structures of all available MST kinase domains (MST1, 3 and 4) and a homology model (MST2) to derive information integrating structure and function.

## Results and Discussion

### Overall structures: kinase domain

**Figure 1:**
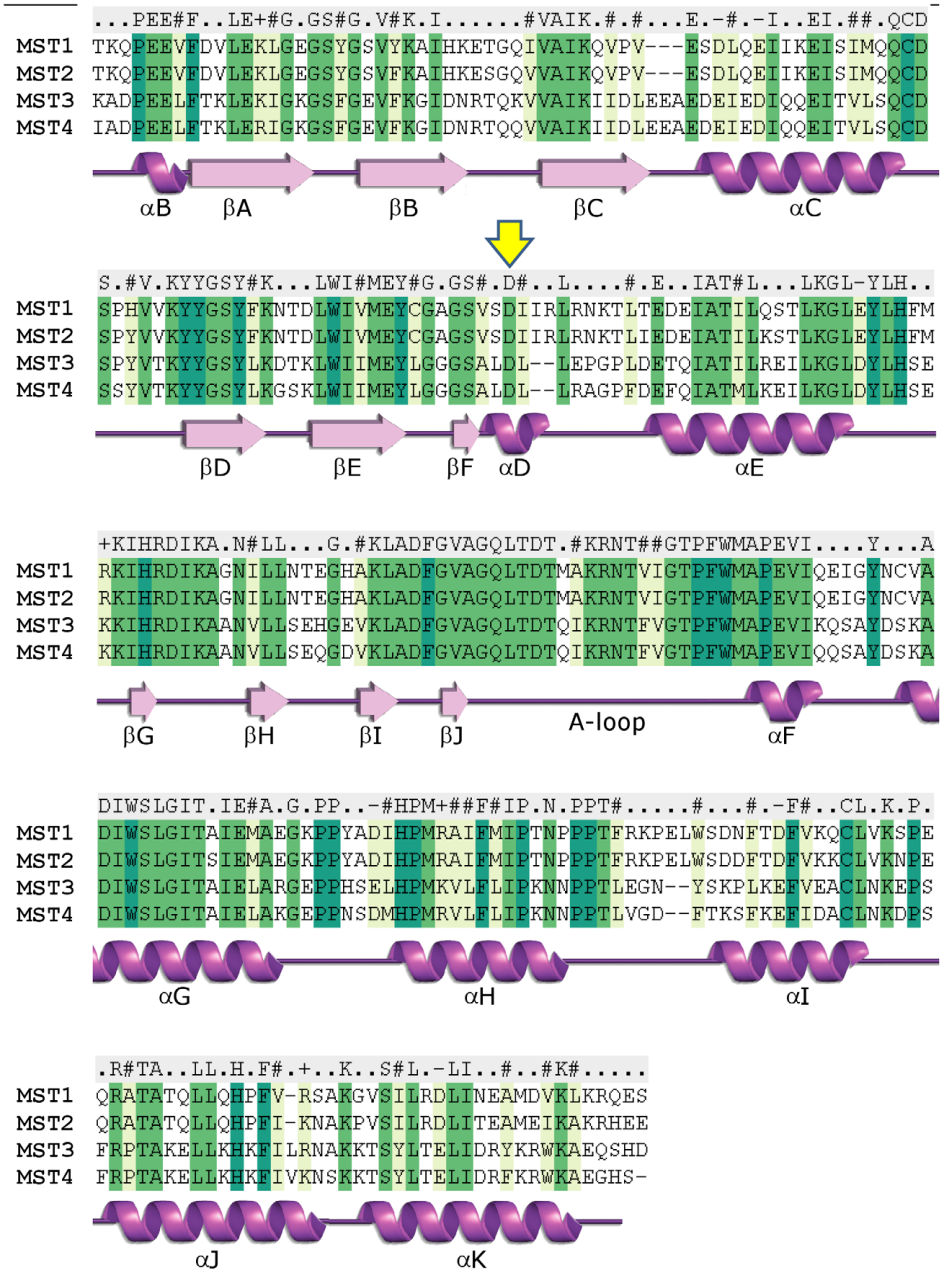
Alignment of human MST kinase domains. The secondary elements shown in the alignment were derived from the structure of MST4 (PDB ID 3GGF). The conserved Asp residue position that is involved in the coordination of ATP via its ribose oxygen atoms is marked with a yellow arrow.

**Figure 2:**
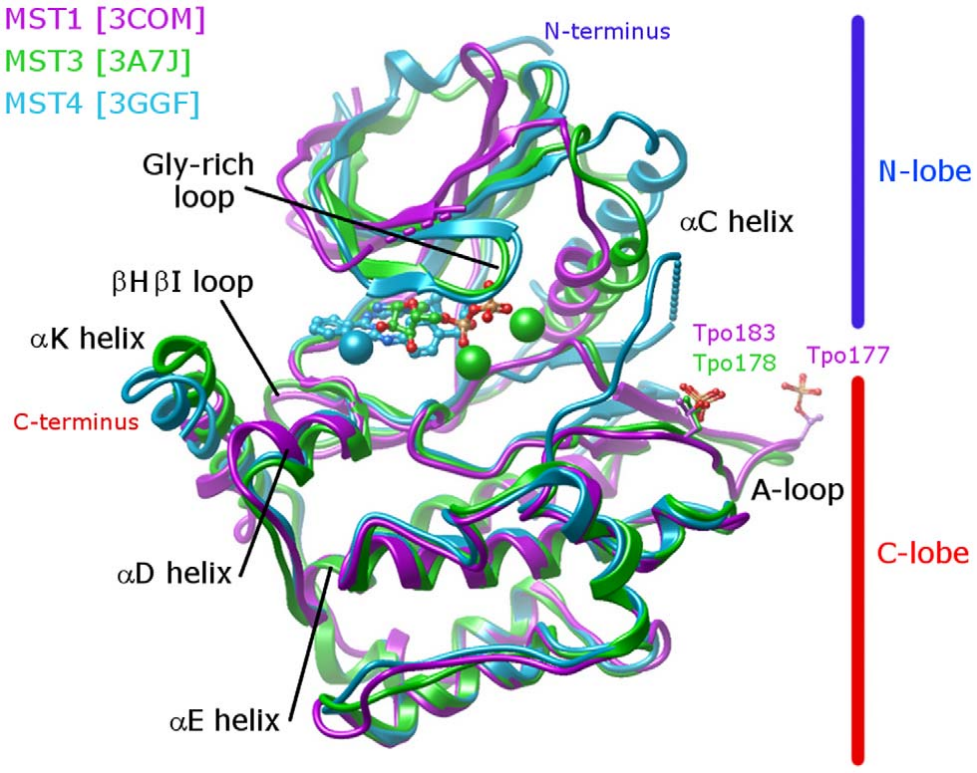
Overall structure of the MST kinase domains. Superimposition of the C-lobe from the three MST structures: Active apo MST1 (magenta, PDB ID: 3COM); Active MST3 (green, PDB ID: 3A7J) in complex with ADP (sticks) and Mn^2+^ ions (spheres); Inactive MST4 (blue, PDB ID: 3GGF) in complex with a quinazoline inhibitor (sticks) and Cd^2+^ (sphere). Phosphorylated threonine residues (Tpo) are shown as sticks.

The overall structure of the kinase domains of MST1, MST3 and MST4 are very similar, as expected for their high degree of sequence conservation, epitomized by 90% identity between the kinase domains of MST3 and MST4 (Figure 1). Nevertheless, structural differences resulted from differential phosphorylation states, complex with ligands and crystal forms provide information on the structural plasticity and regulation of this group of kinases (Figure 2).

The structures of MST1 and MST3 used for comparison with our experimental structure of MST4 are available in the protein data base whereas additional structures of MST3 have been reported recently [44].

The crystal structure of MST1 (PDB ID: 3COM) has been determined without bound cofactor (apo structure) whereas MST3 (PDB ID: 3CKX) has been co-crystallised with a non-selective inhibitor staurosporine. Both MST1 and MST3 structures assume an active conformation, with well-defined activation loops (A-loops) and the canonical salt-bridge between Lys59 and Glu73 (MST1 numbering), despite the difference in the number of phosphorylated sites. The A-loop of MST1 is diphosphorylated (Thr177, Thr183 - UniProt numbering for Q13043) while that of MST3 is monophosphorylated in the canonical position (Thr190 - UniProt numbering for Q9Y6E0-1; numbered as Thr163 in the structure PDB ID: 3CKX). The crystal structure of MST4 (PDB ID: 3GGF; data collection and refinement statistics in table 1) has been solved in complex with an ATP mimetic quinazoline inhibitor. Interestingly, this structure has captured MST4 in its inactive form with a partially disordered, unphosphorylated A-loop. Finally, the conformations adopted by the αC-helix from MST4 also differ from those seen in the structures of the other two MSTs.

**Table 1:** Data Collection and Refinement Statistics.

Complex	MST4-quinazoline
PDB accession code	3ggf
Wavelength (Å)	0.8800
Spacegroup	P2_1_2_1_2_1_
Unit cell dimensions	a = 65.92, b = 91.25, c = 108.97 Åα = β = γ = 90.0°
Resolution range^a^ (Å)	46.78-2.35 Å (2.48-2.35)
No. unique reflections^a^	28094 (4028)
Completeness^a^ (%)	100.0 (100.0)
Average I/σI^a^	9.0 (2.0)
R_merge_ a	0.139 (0.849)
R_pim_ a	0.072 (0.432)
Redundancy^a^	4.6 (4.6)
***Refinement***	
No. Atoms P/L/O^b^	4295/58/153
R_cryst_/R_free_	0.219/0.277
rms. bond length^c^ (Å)	0.016
rms. bond angle^c^ (°)	1.50
B-factor P/L/O^b^ (Å^2^)	21.8/24.5/32.2
B-factor, Wilson plot (Å^2^)	35.8
***Molprobity***	
Ramachandran favour	96.09%
Ramachandran allowed	100.00%

a Values in brackets show the statistics for the highest resolution shell.

b P/L/O represents protein/ligand/other (water, ion and solvent).

c rms indicates root-mean-square.

### The ATP binding site

**Figure 3:**
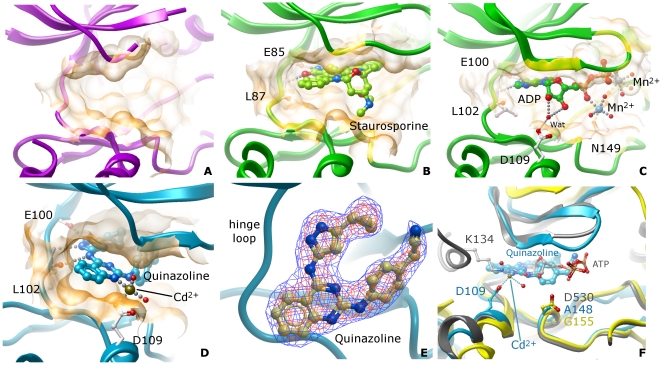
ATP binding site. Different kinases are color coded as follows: MST1: magenta; MST3: green; MST4: blue. Binding site surfaces are represented by semi-transparent orange skins. A. MST1 apo (PDB ID: 3COM); B. MST3 in complex with staurosporine (PDB ID: 3CKX); C. MST3 in complex with ADP (PDB ID: 3A7J); D. MST4 in complex with a quinazoline inhibitor (PDB ID: 3GGF); E. Fo-Fc difference map of the quinazoline inhibitor bound to MST4 (PDB ID: 3GGF). Isosurface contouring levels: 1σ (blue) and 3σ (red). This view is rotated 90° on the X-axis (as compared to the orientation shown in (D) and looks towards the C-lobe; F. Details of the active site of MST4 in complex with a quinazoline inhibitor (blue), superimposed to PAK6 (yellow; PDB ID: 2C30) and TAO2 in complex with ATP (grey; PDB ID: 1U5R).

Regardless of the presence of ligands, the structure of the ATP binding site is very similar for all three MST kinases (Figure 3A–D). In MST3 the bulk of the staurosporine ligand mimics the structure of the ATP adenine rings and sits in the flat cleft between the two lobes. As with MST1, hydrophobic regions above and below the ligand clamp it in place. A minor modification of the immediate pocket is seen in MST3: Ile15 replaces Leu36 in MST1. At the hinge backbone, hydrogen-bonding was observed between two main-chain functional groups; the amide of Leu87 and carbonyl of Glu85, and the staurosporine molecule.

The glycine-rich loop was well ordered in MST3 structures as expected in kinases with a bound ligand. However, residues of the glycine-rich loop have high B-factors in comparison with the rest of the molecule: both structures (in complex with staurosporine in 3CKX and ADP in 3A7J) are lacking the additional stabilization of the glycine-rich loop attributed to the gamma phosphate of the ATP. This can be illustrated by a comparison with the structure of related ser/thr kinase TAO2 which has been co-crystallised with ATP (PDB ID: 1U5R)(Figure 3F).

The binding of the quinazoline inhibitor in MST4 exhibits some interesting features not present in the MST1 or MST3 structures (Figure 3D–F). The presence of a pyrazole group in the ligand introduces the possibility of another H-bond with the hinge region. In addition to the two hydrogen bonds observed in the structure of MST3 in complex with staurosporine, the glutamic acid carbonyl (Glu100) is involved in a third hydrogen bond to the pyrazole group of the quinazoline inhibitor in MST4 (Figure 3D). Additionally electron density data has identified the positions of seven cadmium ions in the asymmetric unit in the crystal structure of MST4, two of which participates in the binding of the ligand in each monomer (Figure 3D). A four-coordinate Cd^2+^ ion (distorted tetrahedral geometry) sits in a location towards the open face of the ligand pocket, close to site occupied by ATP ribose 2′ and 3′ oxygen atoms. It is coordinated by two water molecules, the carboxy group of Asp109, and by a nitrogen atom of the adenine ring. Interestingly, despite the partially disordered A-loop, the geometry of the MST4 ATP pocket has undergone only limited change when compared to MST1 and 3 in the region occupied by the quinazoline ligand.

Superposition of the MSTs with the TAO2 kinase in complex with ATP suggests that the ribose group of ATP does not form any direct interaction with any of the residues lining the ATP binding pocket (Figure 3F). This is further contrasted when other kinases (e.g. PAK kinases) are superimposed – where conserved residues (PAK6 Asp530; PDB ID: 2C30) can interact directly with the oxygen atoms from the ribose group, enforcing the correct geometry for catalysis. Alternatively, an absolutely conserved Asp residue in MSTs (Asp109 in MST4) is found in a position allowing indirect interaction to the ATP ribose. This interaction could be mediated through a water molecule as seen in the structure of MST3 (PDB ID: 3A7J) or a cadmium ion as seen in MST4 (PDB ID: 3GFF). Additionally, in the STE kinase TAO2 (PDB ID: 1U5R) the aspartate residue coordinates a lysine, which in turn interacted with the ribose group. This observation indicates that the positioning of the ribose group through its oxygen atoms by the conserved aspartate requires the presence of a bridging element, which could be charged – a feature that could be explored for future inhibitor design.

**Figure 4:**
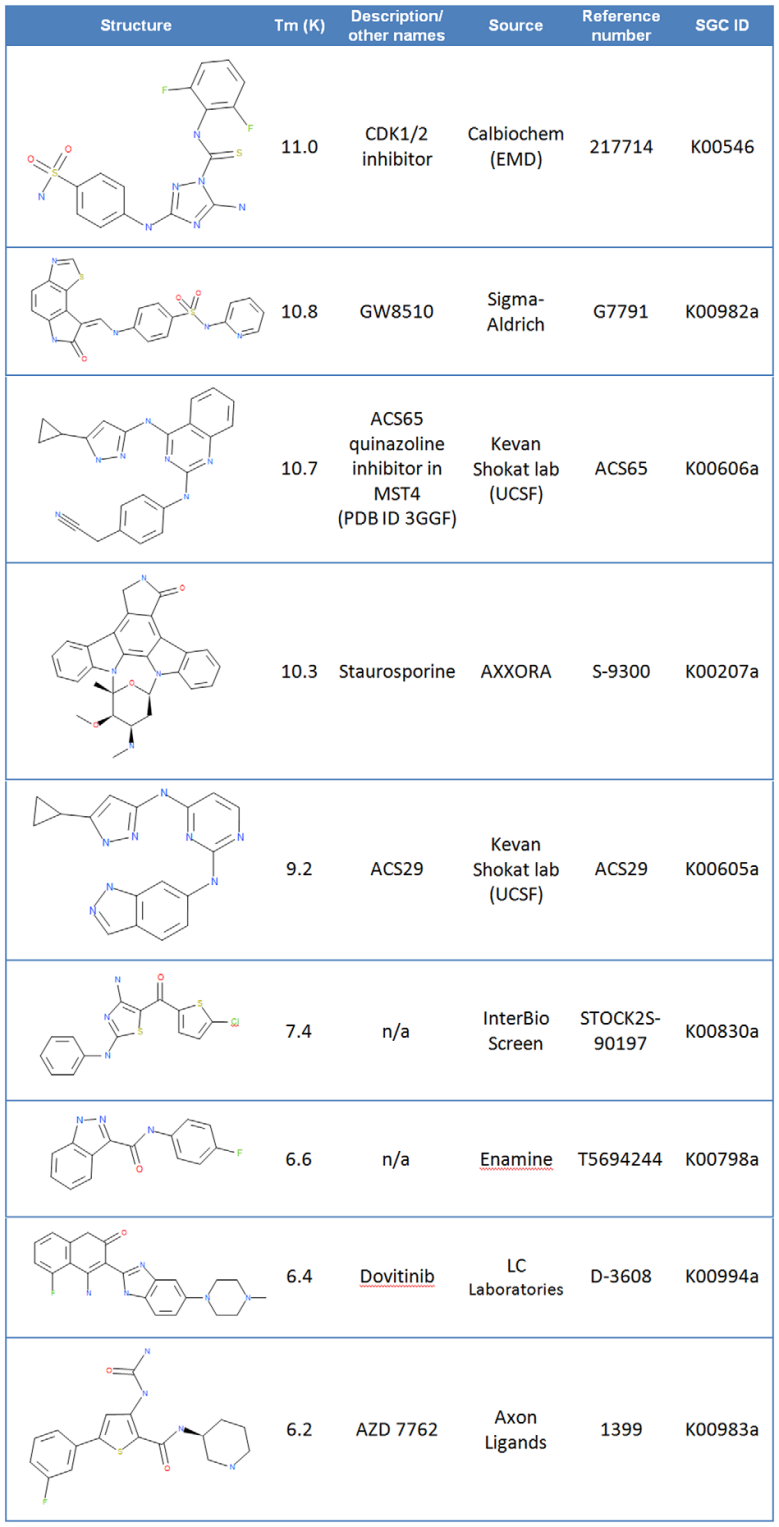
Selected hits from screen of potential MST4 inhibitors against a panel of 1457 compounds.

We have also performed screening of MST4 against a panel of 1457 inhibitors, using thermal stability shift assay (Figure 4). Several compounds have been identified as strong binders, however most are non-specific kinase inhibitors that were previously described in the literature. This is also the case of the quinazoline inhibitor in the structure of MST4. The screening experiments were performed without adding cadmium to the buffer. The observed strong interactions between inhibitors and MST4 suggest that the bridging between Asp109 and ligand could be performed by other molecules, such as water, as observed in the structure of MST3 in complex with ADP (PDB ID: 3A7J).

### α-helix C in MST4 shows an alternative conformation

**Figure 5:**
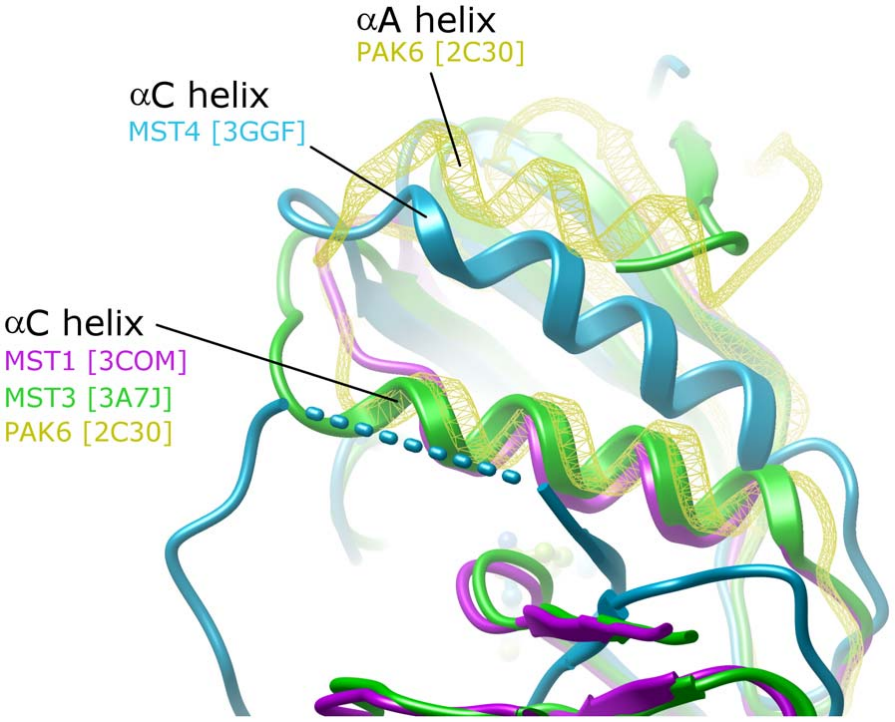
Positioning of αC helix in the structure of three MSTs. The kinase domains were superimposed at the N-lobe. MST1 (magenta), MST3 (green) and MST4 (blue). PAK6 (yellow wireframe) has been added to the superimposition to highlight the position typically occupied by the αC helix, in an active kinase.

The position of the αC-helix in MST1 and 3 is typical for an active conformer of a kinase. Lys59 and Glu73 (MST1) are orientated towards each other with geometry indicative of salt-bridge formation between the residues, with the αC-helix, well-positioned against the N-terminal lobe (N-lobe). The conformation of active MST structures (MST1 and MST3) is similar to conformations observed in other kinase structures especially in PAKs, which also belong to the STE family. It should be mentioned that the helix αA is present in the structures of PAKs and TAO2, stabilizing αC in an active conformation. Thus, the lack of the αA helix in the structures of active MST1 and MST3 does not seem to impact on the αC conformation (Figure 5).

Surprisingly, in the inactive structure of MST4 the αC helix is adopting a ‘swung-up’ conformation, bringing the N-terminal region of the αC helix closer to the N-terminus of the kinase domain. The approximately 13 degrees swinging motion (when compared to the same helix in MST3) allows the A-loop to also swing towards the N-lobe, resulting in the conformation captured in the crystal of MST4.

### Dimerization through activation loop exchange as a model for MST autophosphorylation

The A-loop in both MST1 and MST3 are well-defined and in an active conformation. Interestingly it is diphosphorylated at both Thr177 and Thr183 in MST1 while MST3 is phosphorylated only at Thr178 (structure numbering, PDB ID: 3A7J). Structural superimposition shows that the canonical position (MST1: Thr177, MST3: Thr178) is well conserved, and so are the conformation adopted by the structures of MST1 and MST3 (Cα RMSD: 0.4 Å). In these structures, the canonical phosphorylated sites are coordinated by two conserved arginine residues (MST1: Arg148, Arg181; MST3: Arg143, Arg176).

**Figure 6:**
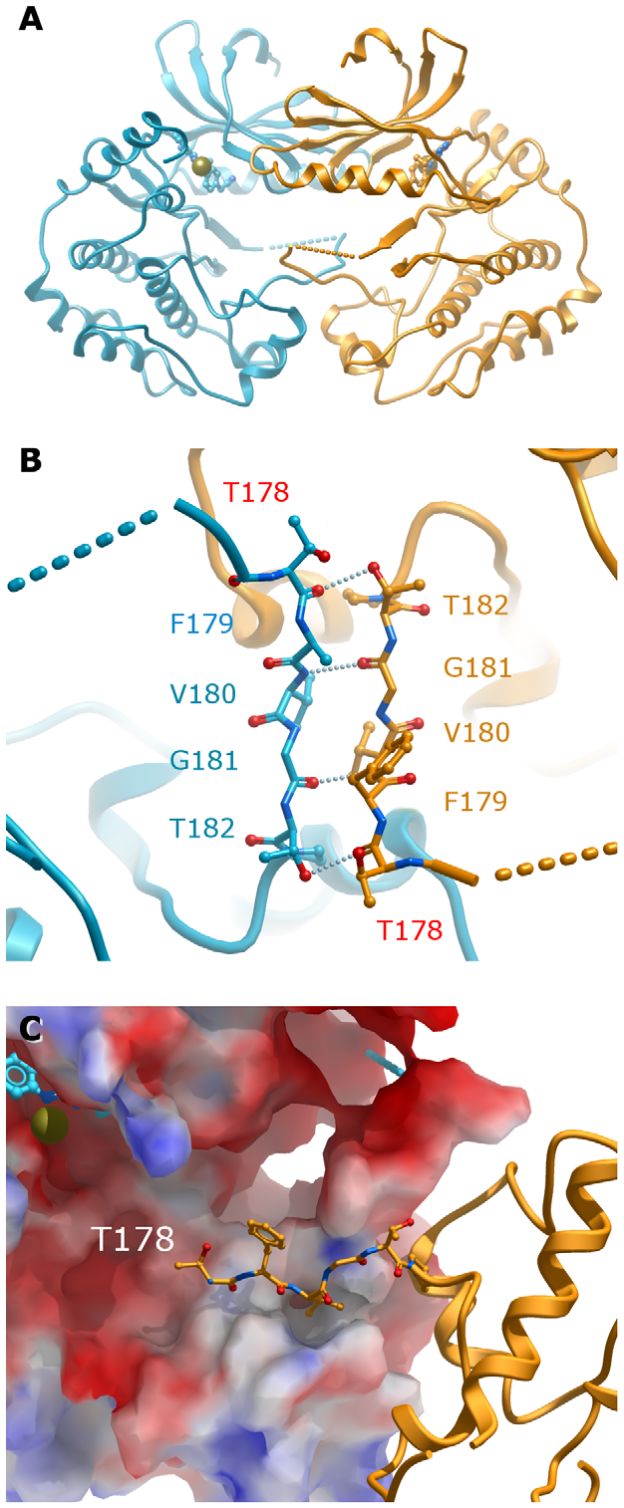
MST4 dimer seen in the asymmetric unit. Chain A is shown in blue, chain B is shown in orange. A. Overall packing. Cadmium ions are shown as yellow spheres, ligands are shown as sticks; B. Molecular interactions seen in the A-loop. Residue Thr178 (to be phosphorylated) is shown with red labels; C. Surface electrostatics potential in the dimerization region – the segment involved in the interaction from the chain B is shown in sticks representation.

In MST4, the unphosphorylated A-loop is positioned in a vastly different orientation when compared to MST1 and MST3 (Figure 2). It is poorly defined, where the visible portions belonging to the N-terminal section of the A-loop are seen adopting a conformation reaching towards the N-lobe, relative to the active form. The C-terminal section is seen extending away from the main core of the kinase domain and reaching into a neighboring kinase molecule, thus correlated with the dimerization seen in this crystal form (Figure 6A).

In the crystal of MST1, the kinase molecules are positioned roughly perpendicular to each other. In such orientation, the active site is out of reach to phosphorylate the A-loop residues Thr177 and Thr183. Similarly, in the structure of MST3, none of the symmetry-related neighbors are found to position the observed phosphorylation site close to the active site, suggesting that the crystal packing of neither MST1 nor MST3 reflect the kinase-kinase interaction positioning necessary for autophosphorylation.

Conversely, as described above, the asymmetric unit of MST4 contains a symmetry-related dimer where the visible C-terminal section of the A-loop of the molecules is contacting each other. Four residues (178-TFVGT-182) of the molecule A are packing against the same residues in molecule B in an anti-parallel disposition, forming a series of hydrogen bonds: each Thr178 main chain carbonyl interacts with Thr182-OG1 from the other molecule; likewise each Val180 main chain nitrogen hydrogen bonds to Gly181 main chain carbonyl from the neighboring molecule. Additionally, the side chains of Val180 are involved in hydrophobic contacts. More critically, the canonical Thr178 to be phosphorylated is found in close proximity to the phosphate transfer site of the neighboring molecule in the dimer (Figure 6C).

The combined observation of the dimer in the asymmetric unit and the A-loop conformation suggest that the kinase domain of MST4 can dimerize in a ‘face-to-face’ fashion. During that stage the inactive and partially disordered A-loop can reach satisfactorily into the substrate binding site of the other kinase in the dimer. In this process Thr178 can be positioned at the catalytic site to be phosphorylated. Similar autophosphorylation mechanism where the A-loops are exchanged in a ‘face-to-face’ kinase dimer has been described before for SLK, LOK and DAPK3 [45], [46].

The observed reciprocal interaction of A-loops in the MST4 dimer implies the availability of a site binding the swapped A-loops. The observed A-loop exchange also creates a distinction between the sequence requirement for the autophosphorylation mechanism and the physiological consensus sequence. Albeit the autophosphorylation consensus has been defined in all human MSTs as KRNT*(V/F)(I/V)GTPFWMAPEVI [9], the physiological consensus has not been determined so far. Ultimately, A-loop swapping allows a single kinase molecule to catalyze two different subsets of residues, which can be explored by designing of peptides (or peptidomimetics) targeting at either the autophosphorylation or physiological consensus binding sites.

While the structure of MST4 supports the A-loop exchange autophosphorylation model, it probably represents an intermediate, pre-catalytic state. The catalysis is dependent on the correct positioning of the αC helix and establishment of the activation salt-bridge, which are not present in the present structure of MST4.

**Figure 7:**
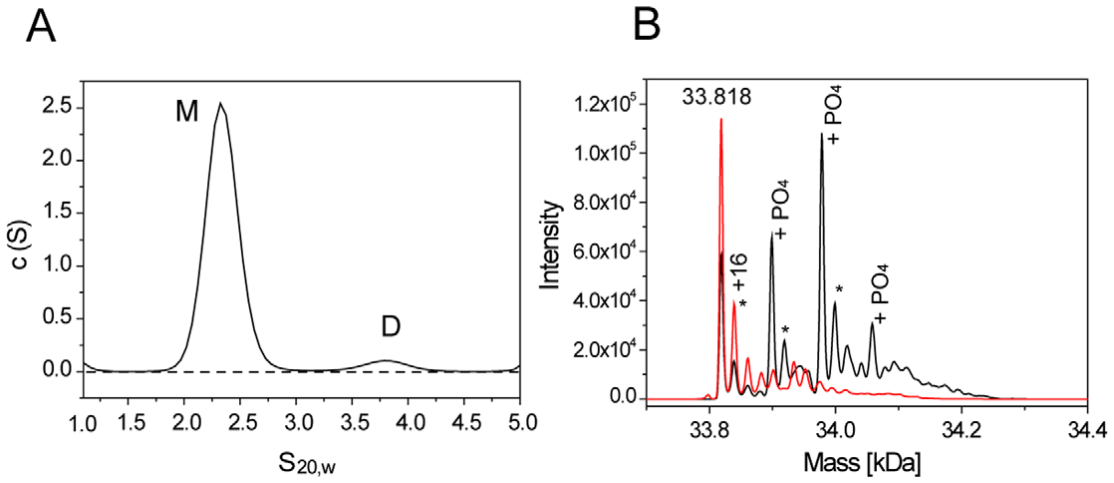
Self association and autophosphorylation of MST4 in solution. A: Self association studied by analytical ultracentrifugation. Shown are sedimentation velocity data. The sedimentation coefficients have been normalized to standard condition. Peaks corresponding to the MST4 monomer and dimer are indicated by “M and D” respectively. B: Autophosphorylation of MST4 studied by ESI-MS. The mass of the unmodified protein (33.818 Da) has been indicated. In addition to the three detected phosphorylation events the protein also oxidized (+16 mass shifts indicated by *).

It is important to emphasize that while autophosphorylation of MSTs seems to involve dimerization, it does not necessarily imply that this is the physiological dimer for the full-length protein. Previous reports have identified a C-terminal dimerization domain in MST1 and MST2, which is not required for kinase activity [47]. Additionally, MST2 lacking the dimerization domain can still autophosphorylate [48]. This suggests that although dimerization driven by the C-terminal domain (at least in MST1 and MST2) might be necessary for other biological/regulatory functions, autophosphorylation *in trans* will probably involve the face-to-face disposition seen in the asymmetric unit of MST4 crystals. We were interested to see if MST4 indeed dimerizes in solution. To this end we performed analytical ultracentrifugation sedimentation velocity experiments. Analysis of the sedimentation data collected on the unphosphorylated protein indicated clearly the presence of MST4 dimers in solution (Figure 7A). In addition, recombinant MST4 are autophosphorylated in solution at three sites, as shown by electrospray mass spectroscopy (Figure 7B).

### C-terminal α-helix K

**Figure 8:**
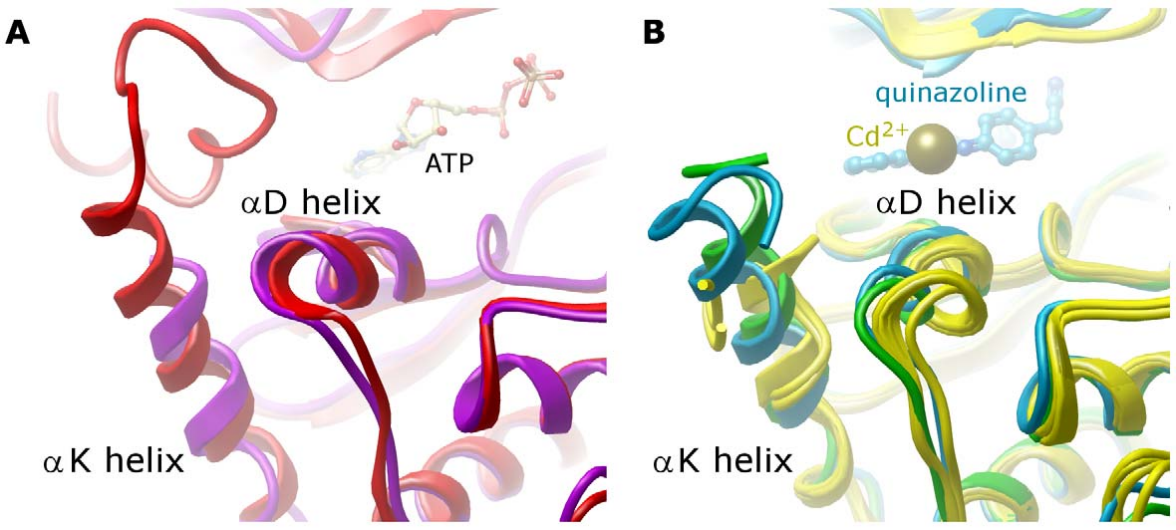
αK and αD conformations for the two subfamilies of MSTs. A. MST1 (magenta) adopts a conformation closer to that seen in TAO2 (red); B. MST3 and 4 (green and blue, respectively) adopts a similar fold to that of PAKs (yellow).

At the C-terminus of the full-length MSTs, we find a regulatory domain, which has been described to be involved in dimerization and not essential to the kinase activity [47]. It can be removed via cleavage with caspase - however the reported cleavage site is located around 30 residues from the last observable residue in the crystal structures [15], [49]. The regulatory domain is connected to the kinase domain via an α-helix (αK) which interacts with the C-terminal lobe (C-lobe) with a multitude of aromatic and hydrophobic interactions. The αK helix spans through the entire height of the C-lobe, packed against a cleft formed by helices αD/αE and the beta hairpin βHβI. Difference in sequences at the C-terminus end of αK between MST1/MST2 and MST3/MST4 (2-residue deletion) alters the αK packing angle. Interestingly, the fold in this region in MST3/MST4 is now similar to that adopted by PAKs whereas the fold adopted by MST1 is comparable to that adopted by TAO2 (Figure 8).

### Surface properties and substrate selectivity

To assess the surface properties, in addition to the three human MST crystallographic structures available at the PDB, we have generated two models by homology: MST2 in the active conformation and MST4 also in the active conformation.

**Figure 9:**
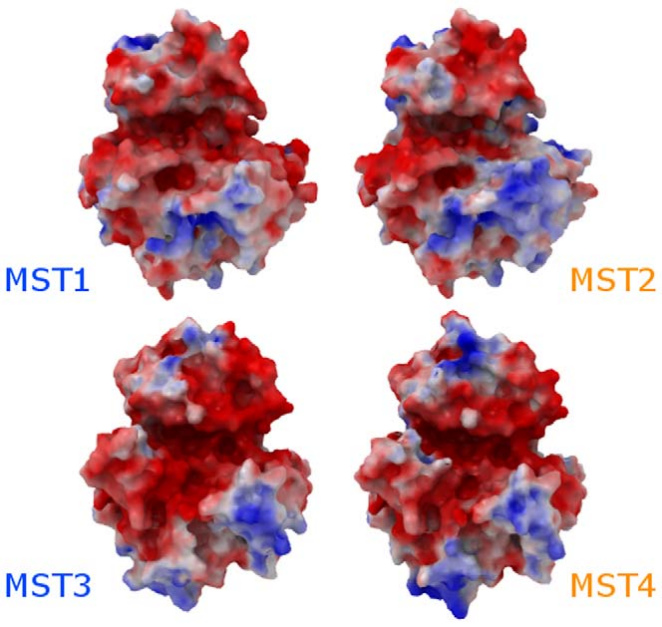
Surface electrostatic potential of MSTs. Crystallographic models are labeled in blue; homology models are labeled in orange. The surface electrostatic potential is represented as a color ramp, ranging from −5 kcal/e.u. charge (red) to blue +5 kcal/e.u. charge (blue).

The distribution of electrostatic potential on the surface of the four human MSTs in their active conformations is remarkably similar despite the occurrence of several patches of low residue conservation (Figure 9). As expected, the surface electrostatic potential of the crystal structure of MST4 (i.e. inactive conformation) was significantly different from those in the active state. This however only highlights the advantages and versatility of the A-loop exchange autophosphorylation model: (i) that the sequence to be autophosphorylated does not need to reflect the kinase's natural substrate selectivity and (ii) that the domain swapping, due to the sequence specific packing, will be also highly specific to the sequence to be autophosphorylated. Additionally, the lack of variability in surface electrostatic potentials reinforces the regulatory role of the divergent C-terminal domain of different MSTs.

## Materials and Methods

### Cloning, Expression and Purification

The MST4 gene corresponding to residues 1–300 was PCR amplified from a Mammalian gene collection library clone and cloned into the expression vector pLIC-SGC1 (SGC) using ligase independent cloning. The MST4 gene sequence of the constructs was verified by DNA sequencing. MST4 protein was expressed in a BL-21(DE3)-R3-pRARE2 cells. The protein was induced by the addition of IPTG to cells growing in LB ampicillin media. Cells were harvested following overnight growth and were stored as cell pellets at −20°C. Approximately 5 grams of cell pellet was resuspended in 25 ml of 50 mM HEPES pH 7.5, 300mM NaCl, 20 mM imidazole. The resuspended cells were lysed by sonication. The lysate was cleared of DNA and cell debris by centrifugation at 20,000 rpm (4°C). Five (5) ml of 50% Ni-NTA slurry (Qiagen) was applied to a 1.5×10 cm gravity column. The column was equilibrated with 100 ml binding buffer. The lysate was applied to the column and was subsequently washed with a minimum of 50 ml of lysis buffer. MST4 was eluted with 25 ml of lysis buffer containing 100 mM Imidazole. DTT was added to the eluted MST4 protein to a final concentration of 5 mM. The N-terminal his6-tag was removed by the addition of approximately 100 mg of TEV protease and incubated at 4°C overnight. Kinase phosphorylation was removed by λ-phosphatase in the presence of 50 mM MnCl_2_. The protein was concentrated using a 10kDa cutoff ultrafiltration unit and further purified by fractionation on a size exclusion chromatography HiLoad 16/60 Superdex 200 column in 50 mM Hepes, pH 7.5, 300 mM NaCl, 5 mM DTT. The protein was diluted to 50 mM HEPES pH 7.5, 50 mM NaCl and further purified by fractionation on a MonoQ column using a 50 mM to 1 M NaCl gradient. The purity of the protein was confirmed by SDS-PAGE and the correct molecular weight was observed by ESI Mass spectroscopy. The collected protein was concentrated by ultrafiltration (10kDa cutoff) to 11.2 mg/ml (stored in 50 mM HEPES pH 7.5, 200 mM NaCl), snap frozen in liquid nitrogen and stored at −80°C.

### Analytical Ultracentrifugation

Sedimentation velocity experiments were carried out on a Beckman Optima XL-I Analytical Ultracentrifuge (Beckman Instruments, Palo Alto, CA) equipped with an AnTi-50 rotor and cells with double sector centerpieces. Protein samples were studied at a concentration of 30 µM in 50 mM HEPES pH 7.5, 100 mM NaCl employing a rotor speed of 45,000 rpm. Radial absorbance scans were collected using absorbance optics at a wavelength of 280 nm in continuous scan mode, in 2 minute intervals. Data were analyzed using the SEDFIT software package [50] whereby differential sedimentation coefficient distributions, *c(s)* distributions, were obtained by direct boundary modeling to Lamm Equation solutions. Sedimentation coefficients, *s*, were obtained by integration of individual peaks in the calculated *c(s)* distributions, after fitting of the frictional ratio, in order to allow these distributions to be corrected for the effects of diffusion. The software package SEDNTERP was used in order to convert the obtained sedimentation coefficient values to the equivalent values in water at 20°C (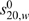,).

### Thermal stability shift assay

Thermal melting experiments were carried out using the Mx3005p real-time PCR machine (Agilent) and employing a protein concentration of 2 µM and inhibitor concentration of 10 µM. Buffer conditions were 10 mM HEPES, pH 7.5, 500 mM NaCl and 1∶1000 dilution of SyproOrange (Invitrogen, CA). The assay and data evaluation were carried out as described [51].

### Crystallization and Structure determination

Crystallization of MST4 was achieved by sitting drop vapor diffusion at 4°C. MST4 (stored in 50 mM HEPES pH 7.5, 200 mM NaCl) was pre-incubated with 1mM quinazoline inhibitor which was added from a 50 mM stock solution in DMSO at a protein concentration of 5.9 mg/ml. The protein was crystallized using 12% PEG 3350, 5mM CdCl_2_, and 0.1M HEPES, pH 7.0. Viable crystals of the complex were obtained after microseeding in a 300 nl drop, mixing protein and precipitant at 2∶1 volume ratio. Diffraction data were collected at Swiss Light Source station X10SA from crystals cryoprotected with a solution composed of mother liquor and 20% PEG 400 and flash cooled to 100°K. The data were processed with MOSFLM [52] and subsequently scaled with SCALA (as implemented in the CCP4 suite [53]). Structure of MST4 was solved by molecular replacement by using the program PHASER [54] and the structure of MST3 (PDB ID: 3CKX) as a model. Initial structure was obtained by automated model building using the Buccaneer program [55] supplemented with an improved phase output from DM (as implemented in the CCP4 suite [53]). Further iterative cycles of manual rebuilding in COOT [56] and refinement with REFMAC5 [57] were performed. Appropriate TLS groups calculated by the TLSMD program [58] were applied to a final round of TLS refinement. Completed structure was verified for geometric correctness with MolProbity [59]. Data collection and refinement statistics are summarized in Table 1.

### Homology modeling

Homology modeling was performed using the ICM software package [60]. The modeling procedure was performed for the full-length of the MST2 kinase domain, since no crystal structure was available. We used the structure of the closely related MST1 (PDB ID: 3COM; sequence identity: 93% for the kinase domain) as the modeling template. Homology modeling, global minimization to solve potential clashes derived from the method (e.g. substitutions, loop modeling/grafting) as well as hydrogen bonding network optimization were performed using the protocols implemented in the program ICM [61].

### Data deposition

The atomic coordinates and structure factors have been deposited with the Protein Data Bank, www.rcsb.org (PDB ID: 3GGF [MST4]).

## Supporting Information

Datapack S1 Standalone iSee datapack - contains the enhanced version of this article for use offline. This file can be opened using free software available for download at http://www.molsoft.com/icm_browser.html.
(ICB)

Text S1 Instructions for installation and use of the required web plugin (to access the online enhanced version of this article).
(PDF)
