# Supplementary material for: Structural Comparison of Human Mammalian Ste20-Like Kinases
Source: PLoS One. 2010 Aug 6;5(8):e11905. doi: 10.1371/journal.pone.0011905 (PMC2920948; doi:10.1371/journal.pone.0011905)
Supplement: Text S1 — Instructions for installation and use of the required web plugin (to access the online enhanced version of this article). (PDF) [file pone.0011905.s002.pdf]

First Time Plugin Installation (to read enhanced article online)

Support: isee@sgc.ox.ac.uk

- Identify and click on the link in the PLoS article. This will take you to the SGC's website with the enhanced iSee version of the article.
- You will reach a page which looks like this:

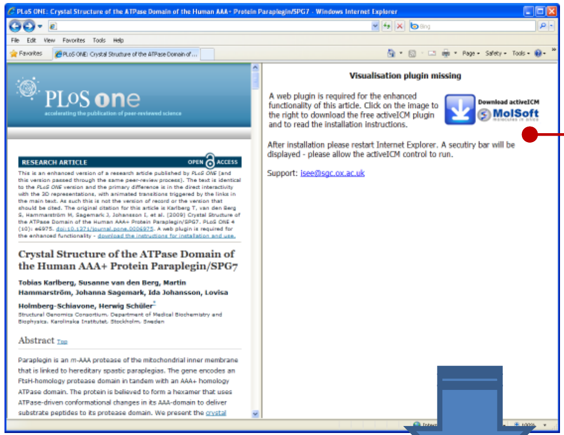

You'll need to install the activeICM plugin in order to see the 3D interactive window – please click on the link from the right window.

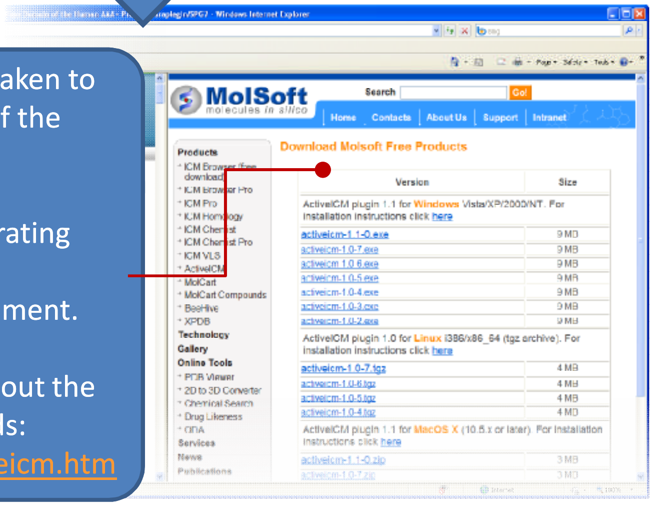

Upon clicking, you'll be taken to MolSoft – the provider of the activeICM plugin.

Please identify your operating system and follow the instructions on this document.

For more information about the plugin and the downloads: www.molsoft.com/activeicm.html

Windows (XP, Vista, Windows 7)

- Download and run the latest installer **(in bold)** and follow the installation instructions
- Close all web browser windows and restart web browser
- **Supported web browsers:** Firefox, IE, Opera or Chrome

Mac OS X

- Download the latest version **(in bold)**

  Firefox: select 'open with DiskImageMounter'; a new window will open – click on the icon to launch the installer and follow the instructions

  Safari: the installer will be launched automatically
- Close all web browser windows and restart web browser
- **Supported web browsers:** Safari 32bit, Firefox

Linux

- Download the latest version **(in bold)**
- `tar xzf activeicm-{version}.tgz` (to unpack the archive)
- `cd activeicmplugin`
- `sh activeicm-plugin-installer` (runs the installer; follow the installation instructions)
- Close all web browser windows and restart web browser
- **Supported web browser:** Firefox

After restarting your browser, open again the link to the enhanced version of the article.

iSee – Quick Reference Chart

There are two main windows in every iSee session – one with text and static images (left) and another which allows interaction with 3D objects (right). The features and behaviours for each window are described below. **Support:** isee@sgc.ox.ac.uk

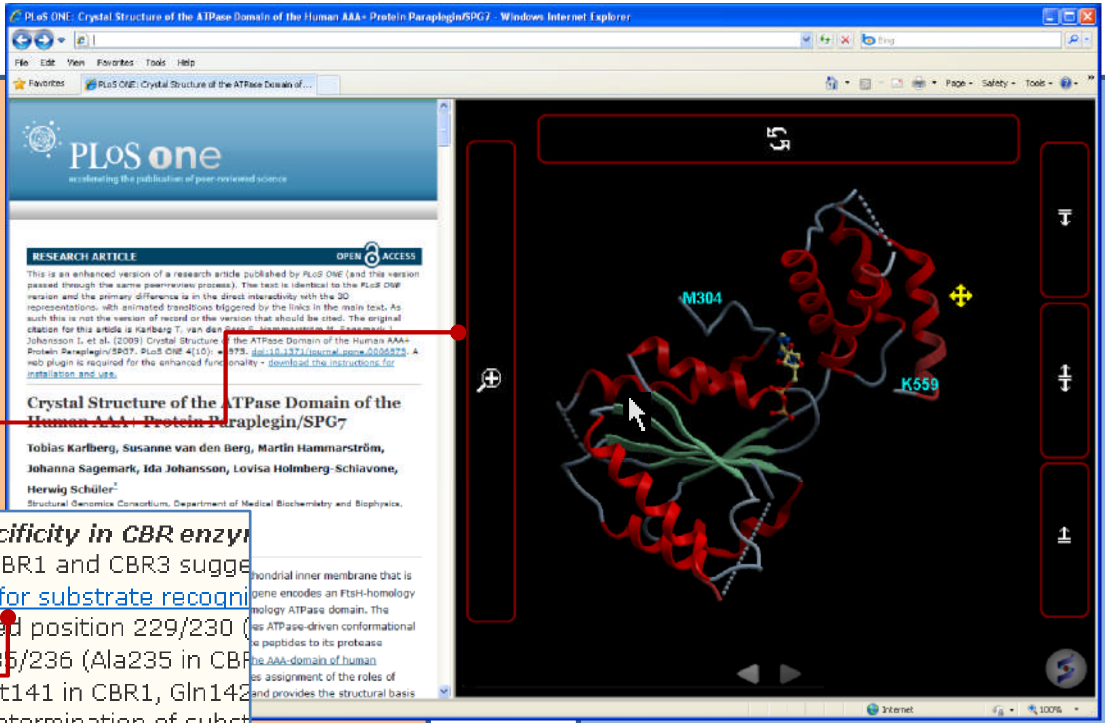

Text and images

- You can resize the browser's main window as well as the ratio between the text/ 3D windows. Place your mouse over the divider, left-click (keep pressed) and drag to resize.

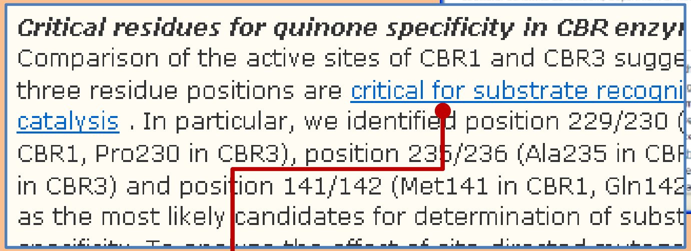
Critical residues for quinone specificity in CBR enzy
Comparison of the active sites of CBR1 and CBR3 sugge
three residue positions are critical for substrate recogni
catalysis . In particular, we identified position 229/230 (
CBR1, Pro230 in CBR3), position 235/236 (Ala235 in CBR
in CBR3) and position 141/142 (Met141 in CBR1, Gln142
as the most likely candidates for determination of subst

- Underlined text is linked to a scene in the 3D window explaining that topic and can be clicked to activate the scene
- Once the scene is shown, you can interact directly with the 3D window to better examine the feature being presented.
- The navigation is not sequential: you can choose to see any scene, anytime, in any order.
- The font size of the text can be changed by using Ctrl+ and Ctrl-
- At any point you can reset the scene to the initial state using the 'reset' link in the text.

Interactive 3D

- Everything displayed in this window can be manipulated using the mouse
- The 3D window has several 'hot-zones' (marked as red boxes in the figure), which provides access to operations that change the scene's view
- Move the cursor into one of these zones to change the dragging function of your mouse (see below)

All the functions described below assumes clicking + holding on the button indicated (i.e. dragging):

move = rotation
up/down = zoom in/out
up/down = zoom in/out
up/down = clip back plane
up/down = clip back+front planes
up/down = clip front plane

Middle button
move = translation

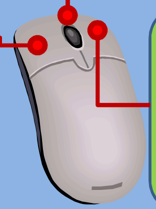

Right button
move = selection (markers appear); select empty zones to remove selection
